# Sap flow of *Amorpha fruticosa*: implications of water use strategy in a semiarid system with secondary salinization

**DOI:** 10.1038/s41598-020-70511-2

**Published:** 2020-08-11

**Authors:** Qiqi Cao, Junran Li, Huijie Xiao, Yuanbo Cao, Zhiming Xin, Benman Yang, Tao Liu, Mutian Yuan

**Affiliations:** 1 College of Soil and Water Conservation, Beijing Forestry University, Beijing, 100083 People’s Republic of China; 2 Department of Geosciences, The University of Tulsa, Tulsa, OK 74104 USA; 3 Beijing Ecological Technology Research Institute, China International Engineering Consulting Corporation, Beijing, 100044 People’s Republic of China; 4 Experimental Center of Desert Forestry, Dengkou Desert Ecosystem Research Station of Inner Mongolia, Dengkou, 015200 People’s Republic of China

**Keywords:** Forest ecology, Plant sciences

## Abstract

*A. fruticosa* (*Amorpha fruticosa* L.) is widely used for revegetation in semiarid lands that undergo secondary salinization. Understanding *A. fruticosa* plants response to soil water and salt stress is essential for water irrigation management and proper revegetation practices. In this study, we measured sap flow, stomatal conductance, meteorological and soil characteristics in an *A. fruticosa* community that recently experienced secondary salinization in northwestern China. Results of our study showed that daytime and nocturnal sap flows averaged 804.37 g·cm^−2^·day^−1^ and 46.06 g·cm^−2^·day^−1^, respectively, during the growing season. Within individual days, the highest sap flow appeared around noon local time and followed a similar pattern of photosynthetically active radiation (*PAR*). Despite the significant effect of meteorological factors on the characteristics of sap flow, our study highlighted that the sap flow of *A. fruticosa* is strongly regulated by the availability of soil relative extractable water (*REW*). The daytime sap flow, which is predominant compared to nocturnal sap flow, was strongly affected by *PAR*, air temperature and vapor-pressure deficit. With water stress in the top 40 cm of the soil (*REW*_0–40 cm_ < 0.4), daytime sap flow displayed a strong relationship with soil water content (*SWC*) (positive) and soil electrical conductivity (*EC*) (negative) in the relatively shallow soil profile (up to 40 cm). For the nocturnal sap flow, our results suggest that in the absence of soil water stress (*REW*_0–40 cm_ > 0.4), the nocturnal sap flow is mainly used to replenish the stem water content and sustain nocturnal transpiration. Under soil water stress, nocturnal sap flow is mainly used to replenish stem water content. The results of our study indicate that it is necessary to shorten the irrigation cycle during the primary growing period (May–July) of *A. fruticosa.* Moreover, in the absence of soil water stress (*REW*_0–40 cm_ > 0.4), *A. fruticosa* can survive well in an saline environment with soil *EC* < 5 mS·cm^−1^.

## Introduction

Secondary salinization is the presence of high salts in soil, which has occurred widely in the semi-arid regions of northwestern China due to the poor irrigation management^1^. To manage both problems of secondary salinization and land degradation, a series of ecological revegetation projects have been implemented in this region in the past few decades. The shrub *Amorpha fruticosa* L. (*A. fruticosa*) is an important species for the revegetation projects because of its drought and salt tolerance^2^. Understanding plant responses to water and salt stress is essential for water irrigation management and proper revegetation practices^3^. However, information about the water use characteristics of *A. fruticosa* remains limited.

The responses of plants to water and salt stress can be evaluated by sap flow, as this is the mechanism of water movement in soil–plant–atmosphere continuum^4^. For many trees and shrubs, daytime sap flow is the most important part of sap flow, but nocturnal sap flow may account for up to 4–69% of the total daily (daytime plus nocturnal) sap flow for a range of species^5^. Daytime sap flow may be affected by a number of meteorological factors^6–9^, including photosynthetically active radiation (*PAR*), vapor-pressure deficit (*VPD*), air temperature (*T*_a_), precipitation (*P*), and wind speed (*u*_s_). Some studies have suggested that nocturnal sap flow is a passive process caused by nocturnal opening of stomata and it may be also affected by *VPD*^10–12^, *u*_s_^13,14^, and *T*_a_^8^. However, other researchers found no significant correlation between nocturnal sap flow and meteorological factors, particularly for trees under severe soil water stress, whose stomata were almost completely closed at night, suggesting that the nocturnal sap flow was used primarily to replenish stem water^15,16^.

Because soil is the primary source of the water that participates in sap flow, soil water can be a critical factor. Some studies found that sap flow increased linearly with increasing soil moisture under a certain threshold level^17,18^. However, others found no constraints of soil water on sap flow in trees, especially for trees with deep roots due to their ability to access more soil water^19,20^. For plants growing in saline conditions, soil salinity can be a vital factor that affects sap flow because water uptake becomes more difficult as the soil water potential becomes increasingly negative^3,21^. However, current findings on the relationship between soil salinity and sap flow are inconsistent. Some indicated that sap flow decreased with increasing soil salinity^3,21^, whereas others found sap flow did not decrease with increasing soil salinity. For example, a study of *Populus euphratica* in an inland river basin in northwestern China showed that sap flow appeared to be unaffected by soil salinity^22^.

In this study, we used *A. fruticosa* as an example to investigate the water use strategy and factors that affect plants’ sap flow under different water and salt conditions in a secondary salinization area in northwestern China. Our objectives were to: (1) examine the daytime and nocturnal sap flow characteristics at monthly, daily, and hourly scales; (2) determine key environmental factors that affect these flows under different soil water and salt conditions; (3) infer the function of the nocturnal sap flow (i.e., whether it is used primarily to support transpiration or to replenish stem water).

## Results

### Environment conditions

#### Meteorology

**Figure 1:**
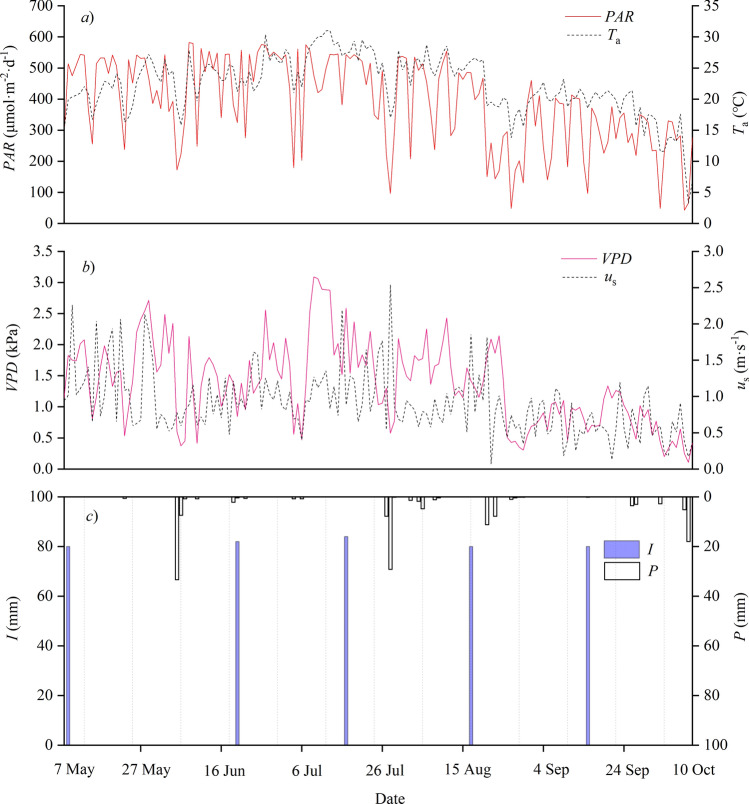
Daily variations in the photosynthetically active radiation (*PAR*), air temperature (*T*_a_), vapor-pressure deficit (*VPD*), wind speed (*u*_s_), irrigation (*I*), precipitation (*P*) during the experimental period in 2017.

Figure 1a shows that during the growth season (May–October), the mean *PAR* was 390.07 μmol·m^−2^·day^−1^, with relative low values in August-October and high values in May–July. Both *T*_a_ and *VPD* followed similar patterns to those of *PAR*, with *T*_a_ ranging from 3.25 to 31.01 ℃ and averaging 22.28 ℃, and *VPD* ranged from 0.11 to 3.09 kPa and averaged 1.36 kPa (Fig. 1a, b). The total precipitation during the study period was 148.2 mm, and most of the precipitation occurred in June–August (Fig. 1c).

### Soil moisture and electrical conductivity

**Figure 2:**
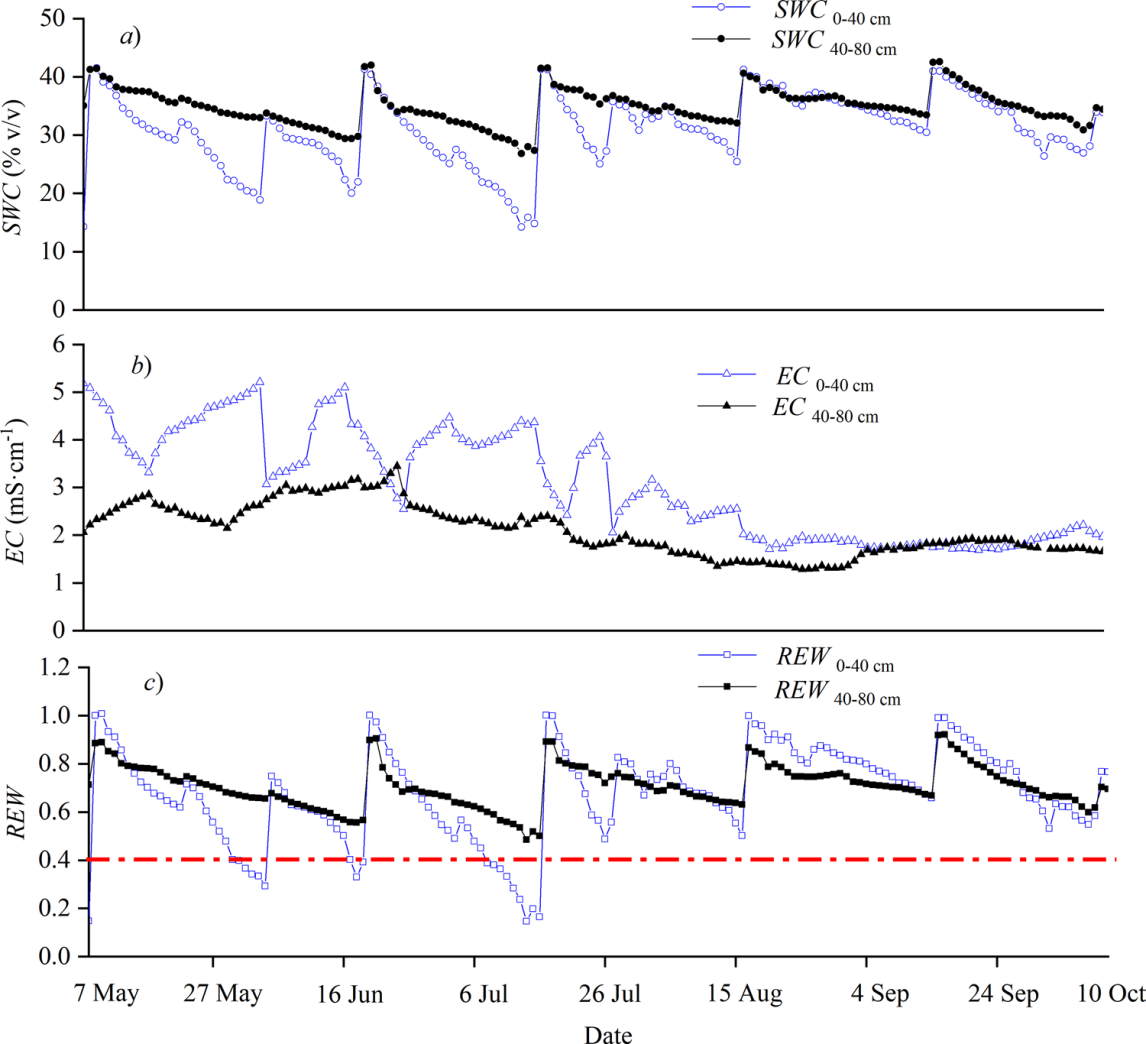
Daily variations in the soil water content (*SWC*), soil electrical conductivity (*EC*), and the relative extractable water (*REW*) at depths of 0 to 40 and 40 to 80 cm during the experimental period in 2017. For *REW*, the horizontal dashed line represents the threshold at which soil water stress begins (*REW* < 0.4).

Values of *SWC* ranged from 14.2 to 41.5% in the 0–40 cm and from 26.8 to 42.6% in the 40–80 cm of the soil profile (Fig. 2a). Field monitoring showed that the top 0–40 cm generally had higher *EC* compared to the 40–80 cm in the soil profile, and the highest *EC* of 5.21 mS·cm^−1^ occurred in the top 40 cm of the soil in early June (Fig. 2b). Soil water stress (*REW* < 0.4) mainly occurred in late May, mid-June, and July in the shallow soil layer, and soil water stress did not occur in the deeper soil layer during the growing period (Fig. 2c).

### Sap flow characteristics at different time scales

#### Monthly

The monthly variations of the mean daytime and nocturnal sap flow during the growing season followed a similar pattern (*r* = 0.99, *P* < 0.01) (Table 1). The maximum mean daytime and nocturnal sap flows occurred in July and June, with values of 1,021.69 and 59.75 g·cm^−2^·day^−1^, respectively, and the minimum mean daytime and nocturnal sap flows occurred in October, with values of 294.64 and 22.76 g·cm^−2^·day^−1^, separately. The monthly mean ratio of nocturnal to total daily sap flow ranged from 5.5 to 8.5%, with the value in October significantly larger than that in other months. The coefficient of variation (*CV*) of daytime sap flow was significantly lower (*P* < 0.05) than that of the nocturnal sap flow, except for July, which had values ranging from 0.08 to 0.32 during the day and from 0.12 to 0.45 during the night.

**Table 1:** Monthly mean daytime and nocturnal sap flows (g·cm^−2^·day^−1^), *CV* of the mean daytime and nocturnal sap flows, and mean ratio of nocturnal to total daily sap flow from May to October.

Month	Sap flow		Ratio (%) of nocturnal to total daily sap flow ± SD	*CV*	
	Daytime ± SD	Nocturnal ± SD		Daytime ± SD	Nocturnal ± SD
May	786.27^ABI^ ± 284.31	47.24^abcII^ ± 7.61	6.34^B^ ± 1.89	0.08^BI^ ± 0.04	0.22^cII^ ± 0.09
Jun	1,019.53^AI^ ± 306.47	59.75^aII^ ± 15.94	6.03^B^ ± 2.26	0.11^BI^ ± 0.04	0.12^bII^ ± 0.08
Jul	1,021.69^AI^ ± 303.52	55.71^abII^ ± 14.23	5.50^B^ ± 1.16	0.13^BI^ ± 0.08	0.13^bII^ ± 0.12
Aug	783.23^ABI^ ± 342.04	42.53^abcII^ ± 14.56	5.64^B^ ± 1.36	0.11^BI^ ± 0.06	0.21^bII^ ± 0.20
Sept	568.77^ABI^ ± 155.23	33.41^bcII^ ± 6.30	5.79^B^ ± 0.82	0.10^BI^ ± 0.07	0.17^bII^ ± 0.14
Oct	294.64^BI^ ± 147.94	22.76^cII^ ± 6.12	8.52^A^ ± 3.89	0.32^AI^ ± 0.24	0.45^aI^ ± 0.30

Different capital and lowercase letters represent significant differences between months for the given parameters (*P* < 0.05), and different roman letters represent significant differences between the given parameters for the same month (*P* < 0.05).

#### Daily

**Figure 3:**
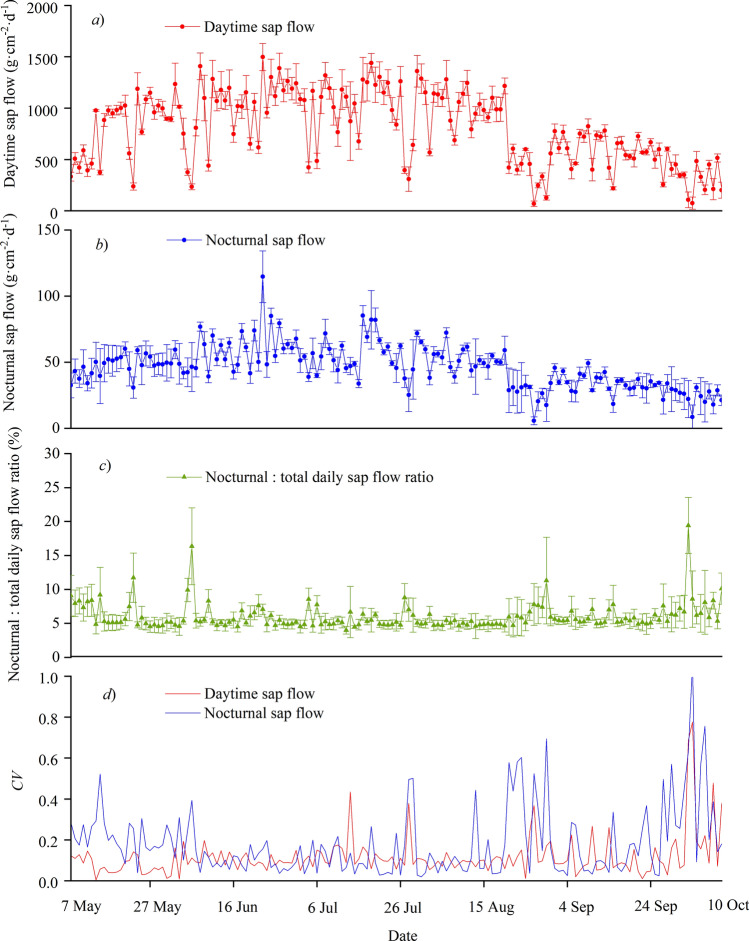
Daytime and nocturnal sap flow (cumulative daily values), ratio of nocturnal to total daily sap flow, coefficient of variation (*CV*) of daytime and nocturnal sap flows for *Amorpha fruticosa* from May to October.

During the growing season, the changes in daytime and nocturnal sap flows showed similar patterns (Fig. 3; *r* = 0.92, *P* < 0.01). Figure 3a shows that June and July, the primary growing season, were also the periods with the highest daytime sap flow. The highest daytime sap flow on 22 June, at 1,497.75 g·cm^−2^·day^−1^. The daytime sap flow in September and October was much lower, with the lowest daytime sap flow on 3 October, at 76.86 g·cm^−2^·day^−1^. The daytime sap flow averaged 804.37 g·cm^−2^·day^−1^ from 7 May to 10 October. In addition, the daytime sap flow was significantly lower (*P* < 0.01) on rainy days.

For the nocturnal sap flow (Fig. 3b), the highest value was 100.77 g·cm^−2^·day^−1^ on 22 June, which agrees with the peak for daytime sap flow. The lowest value was 5.68 g·cm^−2^·day^−1^ on 26 August. The nocturnal sap flow averaged 46.06 g·cm^−2^·day^−1^ from 7 May to 10 October. From May to August, the nocturnal sap flow usually increased after irrigation, and then slowly decreased to the pre-irrigation level.

The ratio of nocturnal to total daily sap flow showed the opposite trend to that for the daytime flow (Fig. 3c; *r* = − 0.63, *P* < 0.01). The ratio of nocturnal to total daily sap flow averaged 6.0% from 7 May to 10 October. Moreover, the ratio was significantly larger (*P* < 0.01) on rainy days. The proportion reached its maximum value of 19.4%, on a rainy day (October 2).

The *CV* of the daytime sap flow was significantly lower (*P* < 0.01) than that of nocturnal sap flow, with values ranging from 0.008 to 0.78 and from 0.02 to 1.06, respectively (Fig. 3d).

#### Hourly

**Figure 4:**
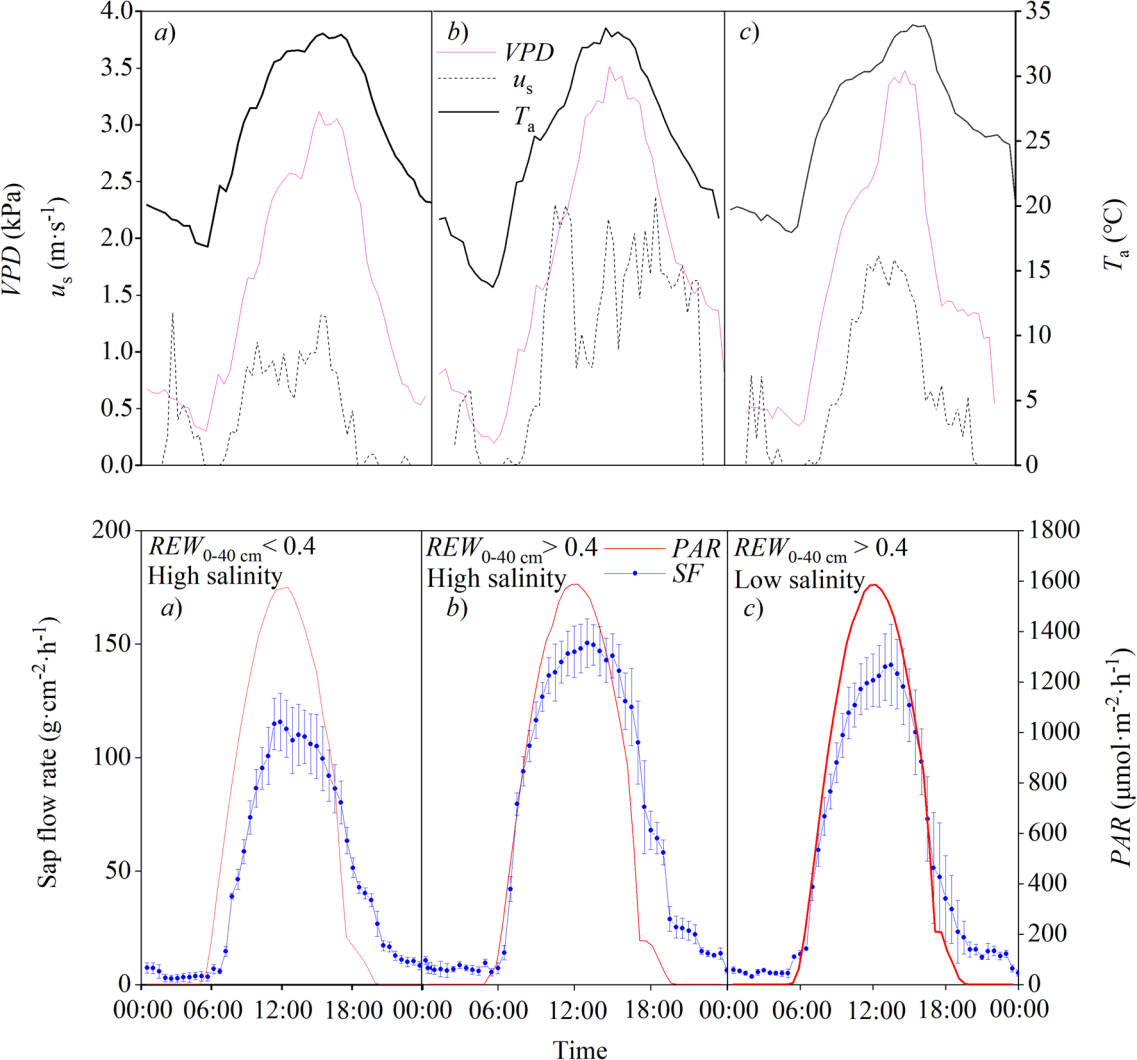
Plots of the hourly changes in sap flow (*SF*), photosynthetically active radiation (*PAR*), vapor-pressure deficit (*VPD*), air temperature (*T*_a_), and wind speed (*u*_s_) for *Amorpha fruticosa* seedlings on three days with typical and similar meteorological conditions but different soil conditions (*REW*_0–40 cm_ represents the relative extractable water in the top 40 cm of the soil; *EC* represents the electrical conductivity). The data are for **(a)** 17 June 2017, a typical day with soil water stress and high salinity (*REW*_0–40 cm_ = 0.33, *EC*_0–40 cm_ = 4.33 mS·cm^−1^, *EC*_40–80 cm_ = 3.15 mS·cm^−1^); **(b)** 22 June 2017, a typical day with no soil water stress and high salinity (*REW*_0–40 cm_ = 0.85, *EC*_0–40 cm_ = 3.32 mS·cm^−1^, *EC*_40–80 cm_ = 3.12 mS·cm^−1^); and **(c)** 19 July 2017, a typical day with no soil water stress and low salinity (*REW*_0–40 cm_ = 0.84, *EC* = 2.62 mS·cm^−1^, *EC*_40–80 cm_ = 2.25 mS·cm^−1^).

For a period of three days, the average *T*_a_, *VPD*, *u*_s_, and *PAR* ranged from 24.42 to 26.13 ℃, from 1.52 to 1.75 kPa, from 0.47 to 1.11 m·s^−1^, and from 532.84 to 544.69 μmol·m^−2^·day^−1^, respectively (Fig. 4).

During the three days, the trends for *PAR* were consistent, with *PAR* > 0 starting at 05:30, and peaking at 12:30, and the change in hourly sap flow strictly followed the change in *PAR* (*r* = 0.95, *P* < 0.01). There was a sharp increase in sap flow after sunrise, with the beginning of sap flow generally lagging behind the onset of *PAR* > 0 by 0.5 to 2.0 h. The sap flow showed a bell-shaped curve with broad peak and no obvious “mid-day break” (i.e., no period with greatly decreased sap flow). However, we found obvious differences (*P* < 0.01) in peak values and in the time lags between the initiation of *PAR* > 0 and that of sap flow between the different soil moisture conditions, whereas the sap flow characteristics showed consistent patterns between the different soil salt conditions. Under soil water stress, the peak daytime sap flow ranged from 95.31 to 115.71 g·cm^−2^·h^−1^, versus from 136.18 to 150.43 g·cm^−2^·h^−1^ in the absence of soil water stress. The onset of daytime sap flow occurred at 07:00 to 07:30 under soil water stress, versus at 06:00 to 06:30 in the absence of soil water stress.

We also observed that nocturnal sap flow was much lower than the diurnal sap flow in three typical days (*P* < 0.01). The nocturnal sap flow averaged 10.35 g·cm^−2^·h^−1^ in the absence of soil water stress, and decreased to 7.73 g·cm^−2^·h^−1^ under soil water stress, whereas the difference was not significant (*P* > 0.05). In addition, the sap flow ranged from 5.21 to 25.40 g·cm^−2^·h^−1^ before the midnight (20:00–24:00), which was significantly higher than that after the midnight (0:00–5:00) (with sap flow ranging from 2.69 to 9.57 g·cm^−2^·h^−1^) (*P* < 0.01).

### Factors affecting sap flow under different soil conditions

The daytime sap flow was significantly and positively correlated with all meteorological factors except *u*_s_ both with and without soil water stress (Table 2). Under soil water stress, there was a significant positive correlation between daytime sap flow and *SWC* in the top 40 cm of the soil. In contrast, the daytime sap flow was significantly and negatively correlated with *EC* in the top 40 cm of the soil under water stress. The daytime sap flow was not significantly associated with *SWC* and *EC* below a depth of 40 cm.

**Table 2:** Correlations (*Pearson’s r*) between daytime and nocturnal sap flows of *Amorpha fruticosa* seedlings and the associated soil and meteorological factors with and without soil water stress.

Sap flow	Condition	*PAR*	*T*_a_	*VPD*	*u*_s_	*SWC*_0–40_	*SWC*_40–80_	*EC*_0–40_	*EC*_40–80_
Daytime	*REW*_0–40 cm_ > 0.4 (*n* = 86)	0.76**	0.76**	0.46**	0.09	− 0.10	− 0.12	− 0.19	− 0.04
	*REW*_0–40 cm_ < 0.4 (*n* = 19)	0.65**	0.64**	0.51*	0.13	0.55*	− 0.24	− 0.46*	0.22
Nocturnal	*REW*_0–40 cm_ > 0.4 (*n* = 86)	–	0.43**	0.40**	− 0.20	0.12	0.04	− 0.14	0.20
	*REW*_0–40 cm_ < 0.4 (*n* = 19)	–	0.20	− 0.16	− 0.34	0.54*	− 0.12	− 0.46*	0.39

Significance levels: *P < 0.05; **P < 0.01. Parameter definitions: *REW*_0–40 cm_, relative extractable water in the top 40 cm of the soil (< 0.4 represents soil water stress); *PAR*, photosynthetically active radiation; *T*_a_, air temperature; *VPD*, vapor-pressure deficit; *u*_s_, wind speed; *SWC*, soil water content; *EC*, electrical conductivity. For the last four variables, numbers refer to the depths (cm) in the soil.

The nocturnal sap flow in the absence of soil water stress was significantly and positively correlated with *T*_a_ and *VPD* (Table 2). Under soil water stress, the nocturnal sap flow was not significantly correlated with any of the meteorological factors, and the nocturnal sap flow was significantly positively and negatively correlated with *SWC* and *EC* in the top 40 cm of the soil, respectively. As same as the daytime sap flow, the nocturnal sap flow was not significantly correlated with *SWC* and *EC* in the soil below 40 cm.

## Discussion

### Characteristics of sap flow

During the growing season (May–October), the daytime and nocturnal sap flows of *A. fruticosa* at monthly and daily scales showed consistent patterns, with high values in May–August and much lower values in September–October. In addition, the daytime sap flow was significantly lower (P < 0.01) in rainy than non-rainy days, which was also reported in many other studies in northwestern China^20,23^. Overall, the nocturnal sap flow accounted for a relatively small proportion (6.0 ± 1.9%) of the total daily sap flow in our study, and this value is well within the range of other studies conducted in similar arid and semiarid ecologies^5,24^.

At hourly scale, the results of our study are consistent with other studies that the peak sap flow decreased under soil water stress^25,26^. Our study further revealed that under soil water stress, the peak values decreased by 22.6% compared with that in the absence of soil water stress. The fact that there is a time lag of 0.5–2 h between the onset of *PAR* > 0 and the onset of sap flow, and the time lag was longer under soil water stress is also well in agreement with other studies that were conducted in similar environments^15,25^. In general, the induced hysteresis, which can be considered as an adaptive strategy by plants, is largely correlated with decreased water uptake due to the increased hydraulic resistance of plants under dry conditions^4^.

We found that the sap flow was significantly higher (*P* < 0.01) in the first half of the night (20:00–24:00) than that in the second half (0:00–5:00) both with and without soil water stress. These results were inconsistent with what reported in Chen et al. (2014)^25^, which demonstrated that the nocturnal sap flow of *Ziziphus jujuba* in the first half of the night was lower than that in the second half. This discrepancy is likely resulted from differences in physiological characteristics between *Ziziphus jujuba* and *A. fruticosa*. when *A. fruticosa* plants experience transpiration process during the daytime, a larger water potential difference will be produced between the roots and the soil during the first part of the night, leading to increased water uptake and a higher nocturnal sap flow rate. In the second half of the night, the water potential difference between the soil and the roots decreases gradually as a result of this increased water uptake, and eventually the sap flow rate decreases^27^.

### Factors affecting sap flow

Some studies indicated that *PAR* and *VPD* are the dominant factors that affect daytime sap flow both with and without soil water stress^3,25^. We found a significant and positive correlation between daytime sap flow and *PAR*, *T*_a_, and *VPD*. Moreover, *PAR* had the strongest effect, followed by *T*_a_ and then *VPD*, both with and without soil water stress. Whereas the nocturnal sap flow was significantly correlated with *T*_a_ and *VPD* only in the absence of soil water stress.

**Figure 5:**
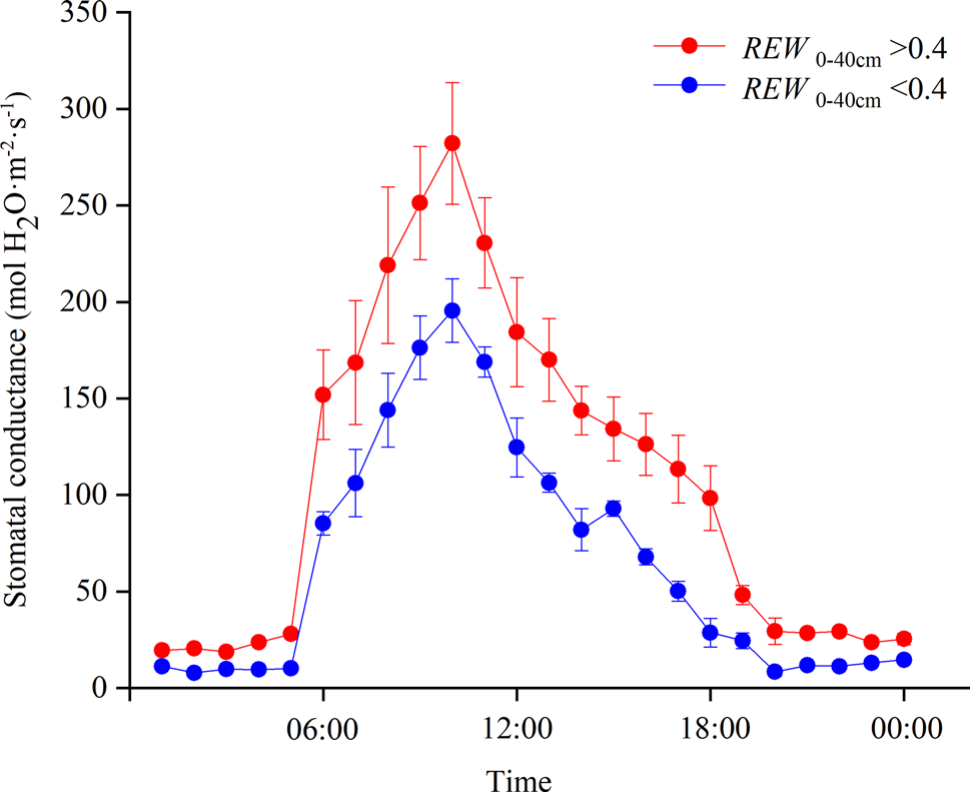
Changes in leaf stomatal conductance of *Amorpha fruticosa* during the day as a function of the relative extractable water (*REW*) in the top 40 cm of the soil: *REW*_0–40 cm_ < 0.4 data from 17 June (soil water stress, with *REW*_0–40 cm_ = 0.33, *EC*_0–40 cm_ = 4.33 mS·cm^−1^, *EC*_40–80 cm_ = 3.15 mS·cm^−1^), *REW*_0–40 cm_ > 0.4 data from 22 June (no soil water stress, with *REW*_0–40 cm_ = 0.85, *EC*_0–40 cm_ = 3.32 mS·cm^−1^, *EC*_40–80 cm_ = 3.12 mS·cm^−1^).

Our study further revealed that under soil water stress (*REW*_0–40 cm_ < 0.4), daytime and nocturnal sap flows of *A. fruticosa* were both significantly and positively affected by soil moisture in the shallow soil layer (0 to 40 cm). However, there was no significant and positive correlations between sap flow and soil moisture in the absence of soil water stress. Chang et al*.* (2014)^17^ and Chen et al*.* (2014)^25^ also found that the sap flow increased with increasing soil moisture but remained almost constant above a certain threshold level. This can be explained by two observations: (i) During the drying progress, shallow soil layers dry out first and the plant may not have access to deeper soil water through their shallower root system, therefore, the hydraulic conductivity of plant will be at a lower level, leading to lower sap flow, and (ii) Plants under soil water stress reduce their stomatal conductance, which would decrease the driving force for water transport through the plant^28^. Moreover, we also found that the leaf daytime and nocturnal stomatal conductance was significantly lower (*P* < 0.05) under soil water stress (Fig. 5). However, the effect of soil moisture in the deep soil layer (40 to 80 cm) was smaller. This result may be explained by the fact that fine roots of *A. fruticosa*, which are primarily responsible for water absorption, are concentrated within the top 40 cm of the soil profile (Fig. 7).

In our study area, under soil water stress condition, with *EC* ranging from 3.89 to 5.21 mS·cm^−1^, we found that the daytime and nocturnal sap flow of *A. fruticosa* decreased significantly with increasing soil salinity. In the absence of soil water stress, soil *EC* had no significant effect on daytime or nocturnal sap flow when *EC* was in the range of 1.89 to 5.08 mS·cm^−1^. This can be attributed to the combination of lower *SWC* and higher *EC* under soil water stress, which would together reduce the soil water potential^28^, and make it more difficult for the plant to take up sufficient water to meet its needs.

### What is the function of the nocturnal sap flow?

Nocturnal sap flow can be caused by nocturnal stomatal conductance, by replenishment of stem water, or by a combination of these effects^5^. Some studies found that stomata are closed completely at night^29,30^, however, Rosado et al*.* (2012)^10^ indicated that weak nocturnal stomatal conductance might result from low levels of nocturnal transpiration. In our study, the stomatal conductance of living leaves was measured for two days (one with and one without soil water stress), the leaf nocturnal stomatal conductance was significantly higher (*P* < 0.05) in the absence of soil water stress (Fig. 5). Other studies also reported that when the nocturnal sap flow sustained nocturnal transpiration, nocturnal sap flow is strongly affected by the meteorological factors^10,31^. Results of our study confirmed that the nocturnal sap flow increased significantly with increasing nocturnal *VPD* and *T*_a_ in the absence of soil water stress. However, under soil water stress, nocturnal sap flow was not affected by the meteorological factors (Table 2).

**Figure 6:**
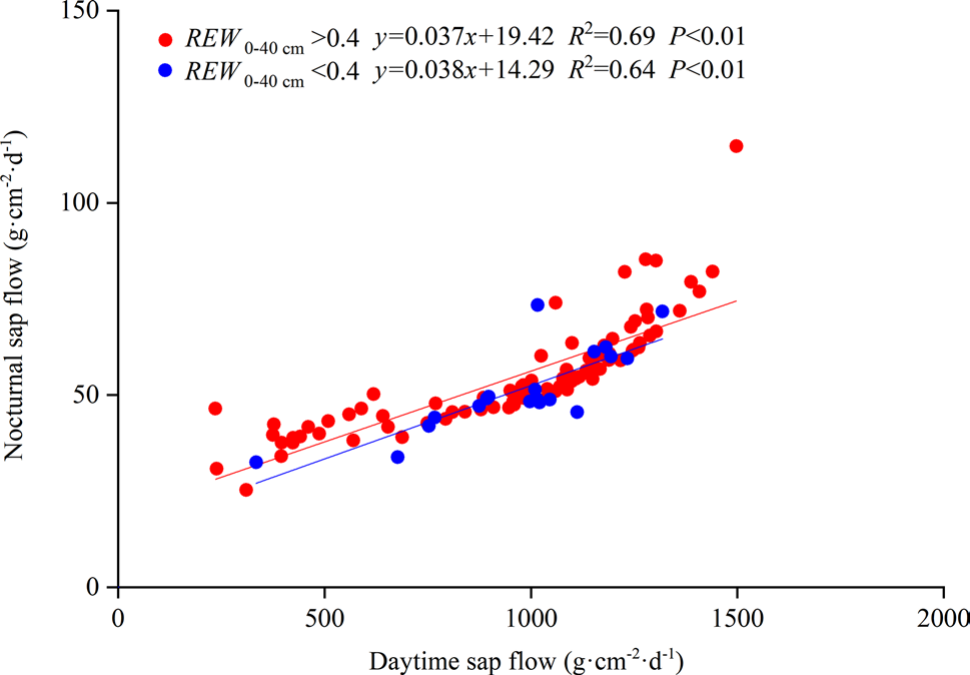
Regression results for the relationships between daytime and nocturnal sap flow under different soil conditions for *Amorpha fruticosa* seedlings during the 2017 growing season. *REW*_0–40 cm_ is the relative extractable water in the top 40 cm of the soil, with a value < 0.4 representing soil water stress.

On the other hand, the nocturnal sap flow can replenish stem water instead of sustaining transpiration, especially following a large daytime sap flow caused by high transpiration^16,32^. In this case, the nocturnal sap flow would increase significantly with increasing daytime sap flow^32^. We found significant positive relationships between nocturnal and daytime sap flow, both with and without soil water stress (Fig. 6)*.* Therefore, we argued that in the absence of soil water stress, the nocturnal sap flow is mainly used to replenish the stem water and sustain nocturnal transpiration, whereas under soil water stress, the nocturnal sap flow is mainly used to replenish the stem water content.

## Materials and methods

### Study site

The study site is located in the Shuxin Forest Farm in Qingtongxia City (38°1′44″ N, 105°56′36″ E; 1,139 m a.s.l.), in Ningxia Hui Autonomous Region, northwestern China. This region lies in the irrigation area of Yinchuan Plain along the upper reaches of the Yellow River. The groundwater level ranges from 0.8 to 1.0 m. The annual average temperature is 8.5 ℃ (with mean monthly temperatures ranging from − 6.5 °C in January to 25.6 °C in July), and the annual rainfall is 175.9 mm.

In 2016 and 2017, this area was flood-irrigated with about 80 mm of fresh water every month from May to September. In April 2016, *Amorpha fruticosa* seedlings were planted in the study area, and these seedlings had an average basal stem diameter of 10 mm and height of 1.0 m. A preliminary soil core analyses showed that soils in the study area on 3 May 2017 (before the first irrigation in 2017) were strongly basic with *pH* > 8 to the depth of 80 cm. The soil electrical conductivity (*EC*) was relatively high in the top 40 cm of the soil and decreased with depth. And the soil is generally silt loam with > 70% of silt in the 0–60 cm soil layers and gradually becomes sandy at the depth of 60–80 cm in the soil profile (Table 3).

**Table 3:** Basic physical and chemical properties of the soil at the study site.

Depth (cm)	*pH*	*EC *(mS cm^−1^)	Bulk density (g cm^−3^)	Wilting point (% v/v)	Field capacity (% v/v)	Soil particle content (% w/w)		
						Sand	Silt	Clay
0–10	8.24 (0.14)	5.87 (0.27)	1.45 (0.03)	9.79 (0.03)	42.06 (0.55)	28.96 (0.15)	70.00 (0.99)	1.04 (0.04)
10–20	8.45 (0.07)	4.91 (0.44)	1.59 (0.03)	9.89 (0.06)	40.91 (0.35)	15.58 (0.22)	81.24 (2.05)	2.18 (0.11)
20–40	8.22 (0.09)	4.03 (0.07)	1.58 (0.03)	9.09 (0.25)	40.86 (0.27)	4.89 (0.10)	92.45 (1.63)	2.66 (0.07)
40–60	8.15 (0.08)	2.71 (0.05)	1.55 (0.03)	7.81 (0.15)	43.07 (0.18)	18.03 (0.17)	79.80 (1.64)	2.17 (0.06)
60–80	8.08 (0.07)	2.54 (0.06)	1.39 (0.01)	10.83 (0.27)	47.76 (0.18)	49.08 (0.20)	16.18 (0.24)	33.74 (0.07)

Values in the parentheses represent standard deviations.

### Field measurements

#### Sap flow measurements

In May 2017, we chose a 6 m × 12 m field plot with 24 *A. fruticosa* seedlings. These plants have a basal diameter ranging from 9 to 27 mm, canopy height of 0.87 to 1.92 m, and the canopy coverage of about 45%. Three representative plants were selected to conduct the sap flow measurements (Table 4). The sap flow of these trees was monitored continuously from 7 May to 10 October using a heat-balance stem sap flow system (Dynamax, Houston, TX, USA). The sap flow gauges were installed on the plant’s main stem, approximately 30 cm above the soil surface. The sap flow data were recorded at 20-s intervals and then averaged every 30 min. The nocturnal sap flow measurements started at the time when the PAR decreased to 0 and ended when it rose to above 0. We used total daily sap flow to represent the sum of the daytime and nocturnal flows, and we reported daytime sap flow when *PAR* > 0. The sap flow rate (*SF*) was calculated as follows:

1 $$ SF = \frac{{\sum\limits_{i = 1}^{n} {F_{i} /A_{si} } }}{n} $$

**Table 4:** Specifications of SGB and the basic properties of the sample trees.

Type	Measured diameter (mm)	Sapwood area (cm^2^)	Stem basal diameter (mm)	Height (m)	Crown width (cm)		Leaf area (m^2^)
					East to west	North to south	
SGB-10	11.82	1.10	12.86	1.28	48.6	40.2	0.27
SGB-13	14.26	1.60	15.82	1.43	70.1	103.7	0.31
SGB-16	17.88	2.51	21.54	1.71	104.1	109.6	0.39

SGB is the type of the sap flow measurement system.

where *SF* is the sap flow per unit sapwood area (g·cm^−2^·h^−1^, or g·cm^−2^·day^−1^); *F*_i_ is the sap flow of the sample tree stem (g·h^−1^, or g·day^−1^); *A*_s_ is the sapwood area (cm^2^); and n is the number of replication (n = 3).

### Fine root traits

Fine root length density (FRLD) and fine root mass density (FRMD) were measured on 13 August 2017 using the root auger with a coring tube (7 cm diameter × 15 cm length). The sampling points were distributed at 20 cm from the trunk of the sample trees in four directions. The root samples were collected at four depths (0–20, 20–40, 40–60, and 60–80 cm). The fine roots (< 2 mm) were picked out from the soil, and then were washed thoroughly. The root samples were analyzed via a WinRHIZO-EC root analysis system (Regent Instruments, Ste-Foy, Québec, Canada) to determine root length. The clean roots were dried in an oven at a temperature of 75 ℃ for 48 h to determine the dry biomass. The FRLD and FRMD were calculated as the ratios of fine root length (cm) and fine root mass (mg) to soil volume (cm^3^), respectively.

### Meteorological variables

We measured *PAR*, precipitation, *u*_s_, relative humidity (*RH*), and *T*_a_ using a small weather station (HOBO, Onset, MA, USA) located about 100 m away from the study site. All these data were recorded at 1-min intervals. *VPD* was calculated from *T*_a_ and *RH* according to Campbell and Norman (1998)^33^:

$$ VPD = 0.{611}e^{\left[ {{17}.{5}0{2}T{\text{a }}/{ (}T{\text{a}} + {24}0.{97)}} \right]}({1}{-}RH). $$

### Soil variables

Soil water content (*SWC*, % v/v) and *EC* (mS·cm^−1^) were monitored with an EM50 meter (Decagon Devices, Pullman, WA, USA) from 5 May to 10 October 2017, and the data were recorded at 30-min intervals. EM50 probes were installed near the sample seedlings at four depths (10, 30, 50, and 70 cm) in the soil to measure *SWC* and *EC* for the layers from 0 to 20, 20 to 40, 40 to 60, and 60 to 80 cm.

**Figure 7:**
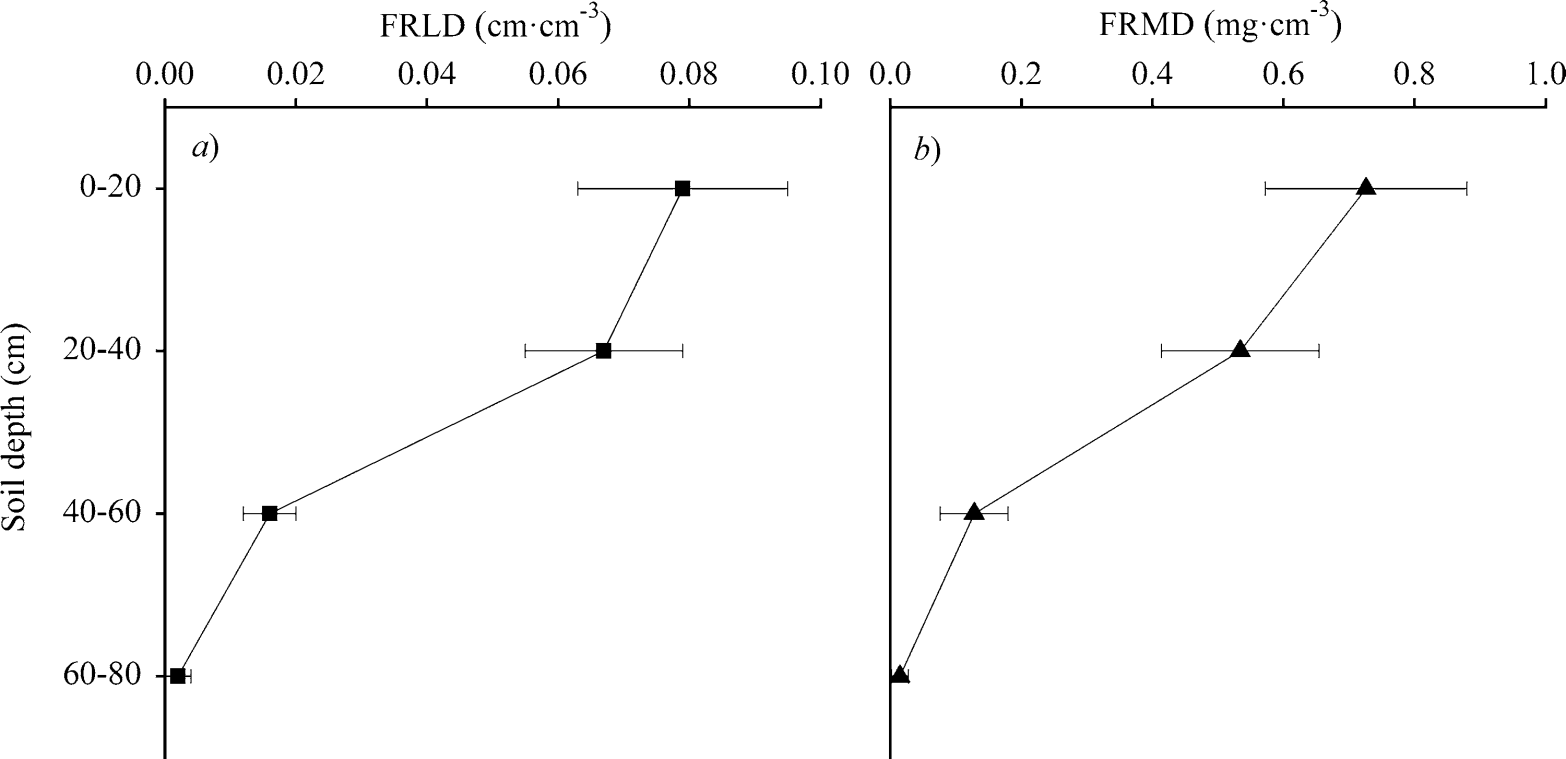
Vertical variations in fine root length density (FRLD) and fine root mass density (FRMD) of *Amorpha fruticosa*.

Figure 7 shows the fine roots of *A. fruticosa* that are responsible for water absorption are primarily located in the top 40 cm of the soil. Therefore, we focused on the *SWC* and *EC* for two layers, namely, 0 to 40 and 40 to 80 cm. The mean *SWC* values were calculated as follows:

2 $$ \begin{gathered} SWC_{{0 - {4}0}} = { (}SWC_{{0 - {2}0}} + SWC_{{{2}0 - {4}0}} {)}/{2} \hfill \\ SWC_{{{4}0 - {8}0}} = { (}SWC_{{{4}0 - {6}0}} + SWC_{{{6}0 - {8}0}} {)}/{2} \hfill \\ EC_{{0 - {4}0}} = { (}EC_{{0 - {2}0}} + EC_{{{2}0 - {4}0}} {)}/{2} \hfill \\ EC_{{{4}0 - {8}0}} = { (}EC_{{{4}0 - {6}0}} + EC_{{{6}0 - {8}0}} {)}/{2} \hfill \\ \end{gathered} $$

### *SWC* classification

During the study period, we used the soil relative extractable water (*REW*) = 0.4 as the threshold to indicate if a soil moisture deficit occurs or water stress develops^20,34^. We defined water stress as *REW* < 0.4. *REW* was calculated using the following Eq. ^20^:

$$ REW = (\theta \, {-} \, \theta_{wp} ) \, / \, (\theta_{fc} {-}\theta_{wp} ) $$

where *θ* is the measured soil water content monitored with the EM50 meter, and *θ*_*wp*_ and *θ*_*fc*_ are the soil water contents at the permanent wilting point and field capacity, respectively.

### Stomatal conductance measurements

We used a steady-state diffusion porometer (SC-1, Decagon Devices, USA) to monitor total daily leaf stomatal conductance on two sunny days (17 June and 22 June). The measurements were conducted on six leaves located at new branches on the sunny side of the selected *A. fruticosa* plants, at a height of 1.0 to 1.2 m above the ground. The stomatal conductance for each plant was obtained by taking the average values of the six leaves. Stomatal conductance was measured every 1 h from 00:00 to 24:00 h.

### Statistical analyses

We used One-way ANOVA to test the monthly mean daytime and nocturnal sap flows, coefficient of variation (*CV*) of the mean daytime and nocturnal sap flows, and mean ratio of nocturnal to total daily (daytime plus nocturnal) sap flow characteristic from May to October according to the least significant difference at *P* < 0.05. We calculated the correlations (*Pearson’s r*) between the mean daytime and nocturnal sap flows and the meteorological and soil factors that affected these correlations to elucidate the key factors that affected the daytime and nocturnal sap flows. As sap flow is also affected by physiological factors^35^. The period (07 May–19 August) is with vigorous growth, 20 August to 10 October is at the later growing stage. In addition, the change of soil water and salt conditions from May to August covered the range of soil conditions during the whole study period. Therefore, we used the data in the period 07 May-19 August to calculate the correlations. We used version 20.0 of the SPSS software (https://www.ibm.com/analytics/spss-software) and version 3.3.2 of the R software^36^ (https://www.r-project.org/) to analyze our data and version 9.0 of the Origin software (https://www.originlab.com/) to illustrate the results of the analyses.

## Data Availability

The datasets used during the current study are available from the corresponding author on reasonable request.
